# Internet usage, frequency and intensity in old age during the COVID-19 pandemic—a case study for Switzerland

**DOI:** 10.3389/fsoc.2023.1268613

**Published:** 2023-10-26

**Authors:** Ronny König, Alexander Seifert

**Affiliations:** ^1^Department of Sociology, University of Zurich, Zurich, Switzerland; ^2^Institute for Integration and Participation, School of Social Work, University of Applied Sciences and Arts, Northwestern Switzerland, Olten, Switzerland

**Keywords:** COVID-19, healthy aging, ICT, internet usage, pandemic, SHARE, Switzerland

## Abstract

**Introduction:**

This study examines the digital divide among older adults in Switzerland within the rapidly evolving digital environment. It investigates changes in internet usage among this population, focusing on the proportion of users, frequency, and the intensity of their internet usage during the COVID-19 pandemic.

**Methods:**

Drawing on Swiss data from the Survey of Health, Aging, and Retirement (SHARE), conducted in 2021, the study analyzes a sample of 1,205 older adults.

**Results:**

The findings indicate a growing proportion of internet users over time. It also highlights that gender differences persist but are decreasing. Notably, around 9% of individuals in this study had never used the internet, while recent users exhibited high activity levels, spending an average of approximately two and a half hours online daily. The study identified age, education, employment, living arrangements, and attitudes toward technology as influential factors shaping internet usage among older adults. Importantly, the COVID-19 pandemic did not have a significant impact on internet adoption among this demographic.

**Discussion:**

These findings shed light on the complex dynamics that shape internet usage among older adults and underscore the need to promote digital inclusion and engagement within this population.

## 1. Introduction

In recent years, we have witnessed the digitalization of numerous everyday activities due to high levels of technological innovation and the rapid diffusion of information and communication technology (ICT; Lechman, 2017). Unfortunately, certain segments of the population, especially older adults, lack access to or proficiency in using this new technology (Seifert and Cotten, 2021). As a result, older adults may be at higher risk of feeling excluded from our digital society (Seifert and Rössel, 2019). Given the rapid evolution and widespread use of technology, individuals today are faced with an ever-changing technological landscape that requires continuous upgrading of devices and software as well as the acquisition of new technology skills (Seifert and Charness, 2022). Therefore, access to current digital technologies, such as smartphones, tablets, smartwatches, smart home devices, and the internet is increasingly becoming indicative of a person’s ability to function effectively in our current day-to-day environment. For instance, in Switzerland, bills were recently transitioned to QR code bills which can now be scanned with an online reader like a smartphone. However, without these technological devices, internet access, and technical skills, it is no longer possible without high effort to pay bills directly to the bank using a payment slip. Although this is a minor example, it illustrates that the exclusion of older people from using technology could also result in their exclusion from society (Seifert et al., 2021). Digital ability and access accumulate over time through individuals’ exposure to digital technologies in institutional, employment, social, and family settings, which provide opportunities for both formal and informal learning (Cotten, 2021). Older adults, in particular, often lack these opportunities. Therefore, they struggle to adopt these modern technologies (Seifert and Cotten, 2021).

## 2. The digital divide in internet use

Even though older adults are increasingly adopting digital technology nowadays, they often exhibit slower adoption rates for the latest technology (Perrin and Atske, 2021). The term “digital divide” refers to the perceived gap between those who have access to the latest information technologies and those who do not, and it is applicable on a global scale. The internet serves as a prime example of modern digital technology and is thus well-suited for illustrating the digital divide. Castells (2003) defines the digital divide as the unequal access to the internet and argues that internet access is a necessary prerequisite for addressing inequalities in a society where dominant functions and social groups are increasingly organized around online platforms.

Although internet use through various devices such as computers, smartphones, and smart televisions has become widespread since the late 1990s, there is still a digital divide based on age and cohort groups regarding individual internet use (Hunsaker and Hargittai, 2018). Younger individuals are currently more inclined to embrace the internet, while adults who did not grow up using these technologies tend to use the internet less frequently. For instance, in the United States in 2021, despite the numerous benefits of the internet and the overall increase in internet usage among the general population, approximately 25% of adults aged 65 and above were not connected to online communities (Faverio, 2022). A representative survey conducted across European Union countries revealed that only 53% of individuals aged 50 years and older used the internet (König and Seifert, 2020). In Switzerland, a survey conducted in 2019 among people aged 65 and older indicated that only 74% of the respondents used the internet (Seifert, 2022). These figures are roughly comparable to internet usage among older individuals in the United States.

From these findings, the question of which older adults use the internet and which do not arises. Aside from sociodemographic characteristics (e.g., age, generation, gender, education, and income) and personal factors (e.g., health, attitudes toward technology, and ICT-related anxiety), environmental circumstances, including the availability of ICT infrastructure, also contribute to the differences in technology usage between the younger and older populations (Hunsaker and Hargittai, 2018; König et al., 2018; König and Seifert, 2020). The significance of sociodemographic and socioeconomic determinants is also emphasized in international literature. For instance, studies such as Scheerder et al. (2017) illustrate how internet use varies among different age groups and individuals with diverse educational backgrounds and financial resources. A recent study by König and Seifert (2020) focused on older adults in Europe (people aged 50 and older) and examined changes over time to their internet usage, including transitioning online and offline, as well as the predictors of such changes at the micro, meso, and macro levels. Respondents who had not used the internet in the 7 days before the baseline but had used it in the 7 days before the follow-up interview were classified as “becoming onliners,” whereas the opposite behavior defined was classified as becoming offline. Overall, a major finding was the low but not negligible percentage of older adults who no longer use the internet. These changes were influenced by shifts in personal resources (such as financial resources), health-related issues (such as health limitations), and social circumstances (such as a loss of social support). Consequently, older adults can experience both gains and losses in technical competency and usage as they age.

## 3. Besides basic access: frequency and intensity of internet use

The change in the digital divide over time and among older adults makes it clear that older people can learn new technologies and that the status of being an offliner is not unchangeable. However, it also means that former onliners can become offliners due to factors such as increasing health problems or a lack of support from their social environment. Therefore, the dichotomy of “user/non-user” often argued by the classic technology acceptance model (TAM; Davis, 1989; Venkatesh and Davis, 2000) should not be seen as a static condition. Instead, it should be seen as a dynamic process in which older people can experience gains and losses. Accordingly, internet access is only the first level of ensuring online participation. Once this level is achieved and the internet is accessible, older adults still tend to use the internet less and have fewer related technical abilities (Hargittai et al., 2019). This phenomenon, known as the second level of the digital divide (Scheerder et al., 2017), indicates that besides access to the internet, it is important to consider what older adults actually do with the internet and how frequently and intensely they use it. Research shows that, compared to younger adults, older adults use the internet less frequently and with lower intensity (Seifert and Rössel, 2019). According to the second level of the digital divide approach, the actual use of the internet is defined in terms of frequency, duration of internet use, and/or the type of activity performed online (van Deursen and Andrade, 2018). In our study, we focused on the frequency and duration (intensity) of internet use among older adults.

“Frequency” of internet use is often measured by the number of times individuals spent online within a year, and a distinction is commonly made between daily use, several times a week, several times a month, and less frequently (Seifert, 2022). Friemel (2016) observed an exponential decline in the frequency of internet use among Swiss older adults after the age of 70, indicating a decreasing trend in internet use within this age group. Therefore, there is a significant difference between older adults who use the internet only once a month and those who use it on a daily basis. In addition to frequency of use, the intensity of internet use is often assessed in media research in general (Nie and Erbring, 2002) by measuring the amount of time spent online per day (i.e., how intensively does the person use the Internet on a normal day). The results of this study revealed that older adults exhibit a diverse range of activities on the internet. For instance, a study conducted in the Netherlands (van Boekel et al., 2017) demonstrated that among individuals aged 65 years and older, the oldest respondents (within a mean age 74 years) spent the least amount of time on the internet. These individuals, referred to as “minimizers,” reported the lowest frequencies of engagement in most internet activities and primarily utilized the internet for traditional purposes like email. On the other end of the spectrum were the “maximizers,” who were relatively younger (within a mean age below 70 years), spent the most time on the internet, and participated in a wide range of online activities. Therefore, it is crucial for research on the digital divide to encompass not only the basic categories of “use” and “non-use” but also the frequency and intensity of internet use as well as factors that predict differences in frequency and intensity.

## 4. The impact of the COVID-19 pandemic on internet use among older adults

The COVID-19 pandemic had a comprehensive and diverse impact on our everyday lives, and internet use played an especially important role in enabling older individuals to communicate, work, and receive healthcare during the pandemic (Seifert et al., 2021). However, the aforementioned digital divide was also evident during the pandemic (Robinson et al., 2020). With social distancing mandates in place in many areas of the world, social interactions were often minimized. One way in which many individuals with digital resources were able to overcome these social distancing mandates was through the use of ICT to maintain contact with their social connections. Although older adults are increasingly bridging the digital divide, a significant portion of them do not use the internet and therefore were not able to benefit from social connections, such as video calls and online meetings, via the internet.

Although internet use can assist older adults in maintaining social interaction, those who do not use the internet or only utilize its basic functions (such as those who only use email, referred to as “minimizers”) may experience social isolation due to their lack of skills and access to digital technology. The challenge of acquiring new technology skills, such as internet use, is particularly significant for older adults, some of whom had to adapt and learn these skills during the pandemic (McClain et al., 2021). Current research indicates that there was only a slight increase in internet usage among residents of long-term care facilities in Austria. This unexpected lack of a “digital push” during the pandemic highlights the existing gaps in research regarding the internet usage patterns of older adults, especially considering the ongoing impact of the pandemic.

## 5. Research questions

As the literature has indicated, numerous studies emphasize the digital usage or non-usage of the internet, particularly among older individuals, and provide explanatory factors for these patterns. However, there has been limited consideration of the COVID-19 pandemic’s context, and a comprehensive differentiation between predictors of Internet use, frequency, and intensity is absent. The present study seeks to address this research gap by utilizing data from Switzerland.

In line with the current state of research, we focused on three research questions:

(1) How did the proportion of older internet users change between 2011 and 2021 as well as in the light of the COVID-19 pandemic?
(2) How frequently and intensely are older adults using the internet during the first year of the pandemic?
(3) What are the predictors for internet use, frequency, and intensity at the micro, meso, and macro levels?

## 6. Data and methods

### 6.1. Data

To address our research questions, this study utilized data from the Survey of Health, Aging, and Retirement (SHARE), which provides standardized information on individuals aged 50 years and older in various European countries. Our main sample consisted of the Swiss subsample from the second COVID-19 survey, conducted between June and August 2021. Specifically, our main variables of interest were derived from the additional drop-off questionnaire, which was administered exclusively to the Swiss sample shortly after the field phase using written questionnaires (for further details, see Börsch-Supan, 2022e). Depending on respondents’ reported internet use in the regular second COVID-19 survey, two different versions of the questionnaire were sent. While most questions were similar in both versions, respondents who indicated internet usage for activities such as emailing, information search, online purchases, or any other purpose at least once since the outbreak of the COVID-19 pandemic received a questionnaire Version A. This version included questions on individual internet usage, usage behavior, and technology experience. On the other hand, respondents who did not report recent internet use received questionnaire Version B, which was designed to gather information on their reasons, concerns, and motivations for not using the internet. In total, out of 1,751 participants from the second COVID-19 survey, 1,566 completed the additional Swiss drop-off questionnaire, resulting in a response rate of 89 percent.

While the Swiss drop-off questionnaire in 2021 includes a range of ICT-related questions, the SHARE generally asks about recent internet use in the later stages of participants’ life based on a single item starting from Wave 4 (2010/12). Although the wording of this question varies to some extent across subsequent waves, comparing them still provides initial insights into general internet use and its variations over time. Therefore, we also incorporated data from the Swiss sample of Waves 4, 5, 6, and 8 (for details, see Börsch-Supan, 2022a,b,c,d) to examine internet use trends over a 10-year period.

### 6.2. Dependent variables

Since the study focused on internet usage, frequency, and intensity, we considered different dependent variables. The first question regarding internet use was introduced in the fourth SHARE wave as follows: “Do you regularly use the World Wide Web, or the internet, for sending and receiving e-mail or for any other purpose, such as making purchases, searching for information, or making travel reservations?” From Wave 5 to Wave 8, the time limit for regular internet use was specified by asking “During the past 7 days, have you used the internet, for e-mailing, searching for information, making purchases, or for any other purpose at least once?” While internet use was not included in the first COVID-19 survey conducted in 2020, the second survey in 2021 included the following question: “Since the outbreak of Corona, have you used the internet, for e-mailing, searching for information, making purchases, or for any other purpose at least once?” All questions contain two answer options, “yes” and “no.”

In addition to capturing this so-called recent internet use, the additional drop-off questionnaire conducted in 2021 for the Swiss subsample covers a wide range of other internet-related items. First, we assessed whether respondents had ever used the internet independently, with response options of “yes” or “no,” based on the question: “Have you ever used the internet without help?” Second, to compare general internet use over time and across different SHARE waves, we examined recent internet use based on the question “In the last 6 months, how often have you used the internet yourself on average?” Respondents could choose from six options: “daily,” “several times a week,” “several times a month,” “less often,” “never,” and “do not know.” We excluded the “do not know” responses and categorized respondents who reported daily usage or usage several times a week as “recent” internet users. Conversely, those who reported less frequent usage (several times a month, less often, or never) were classified as “non-recent” internet users. Third, we used this information with the initial coding to capture internet frequencies on a five-point scale in reverse coding from “never” to “daily.” Respondents defined as internet users in the second COVID-19 survey who answered Version A of the drop-off questionnaire were asked more questions about their daily internet usage. Therefore, we included internet intensity by using two different answers to a question. The question was, “On days when you use the internet, on average, how many hours and minutes do you spend on the internet on those days?” Respondents provided estimates for their private and professional internet use separately. Based on both answers, we calculated the overall intensity and recorded the total and private usage times in minutes. Therefore, we define intensity in terms of the duration of general internet use and did not specifically focus on different internet uses (e.g., e-commerce, social networking). We aim to incorporate intensity as a variable by considering the duration of general internet use in our analyses, as this approach is rarely used as an explanatory variable for older individuals (Hunsaker and Hargittai, 2018). As most of the surveyed individuals were already retired (78%), we analyzed professional internet use separately.

### 6.3. Independent variables

To analyze the patterns of different types of internet use in the later stages of life, we examined basic sociodemographic and economic variables. These include age (measured as a continuous variable in years), gender (differentiating between “men” and “women”), educational level, occupational status, and financial situation. Education was assessed based on the respondents’ level of education according to the International Standard Classification of Education (ISCED). It was categorized into three levels: “low” (ISCED 0–2 indicating (pre)primary and lower secondary education), “medium” (ISCED 3–4 indicating upper and post-secondary education), and “high” (ISCED 5–8 indicating tertiary education). Occupational status was coded binarily as “employed” or “unemployed/inactive.” Income was measured based on the question of whether the household had enough money to make ends meet and split into three categories: “with great/some difficulty,” “fairly easily,” and “easily.”

As health has been found to be associated with psychological well-being and cognitive functioning, we considered the respondents’ self-rated health conditions, ranging from “excellent” and “very good” to “good,” to “fair/poor.” In addition to physical health, we included the shortened and revised Control, Autonomy, Self-realization, and Pleasure (CASP)-12 scale, which is a commonly used measure of quality of life (QoL) among older people (for details on the initial CASP scale, CASP-19, see Hyde et al., 2003; Wiggins et al., 2008). The CASP-12 scale is based on 12 items and has a range of scores from 12 (minimum) to 48 (maximum), with higher scores indicating higher subjective well-being. Considering that personality is known to be associated with social participation (e.g., Roberts et al., 2007), we also incorporated the 10-item Big-Five inventory (BFI-10), which was introduced by Rammstedt and John (2007) and obtained from the seventh wave of the SHARE survey (2011). This established personality inventory measures five dimensions of personality (openness, conscientiousness, extraversion, agreeableness, and neuroticism) with two items for each dimension (for further information on the measurement, please see Börsch-Supan et al., 2019). Each dimension can be expressed in nine different values ranging from “low” (1) to “high” (5) with increments of 0.5.

As living alone can contribute to feelings of loneliness and social isolation, especially during a pandemic when social distancing is necessary to reduce the risk of infection, we examined whether respondents live alone or not. Previous studies have highlighted the significance of regional differences in the adoption of modern technologies (König et al., 2018). These differences can be attributed to factors such as variations in broadband internet availability and diverse needs and habits related to modern technologies based on the degree of urbanization in the area. While general internet availability may not vary regionally within Switzerland (Seifert, 2022), we considered respondents’ living area by distinguishing between “urban” (big cities, suburbs or outskirts, and large towns) and “non-urban” (small towns, rural areas, and villages) places of residence. Given the geographical, linguistic, and cultural proximity of German-speaking Switzerland to Germany and Austria, French-speaking Switzerland to France, and Ticino to Italy, we made the assumption, based on the spillover hypothesis and previous research findings (e.g., König et al., 2018; König and Seifert, 2020; Seifert, 2022), that internet use, frequency, and/or intensity might vary among the three language regions in Switzerland. Although we are unable to include Romansh (Switzerland’s fourth official language) in our analysis, we acknowledged this phenomenon by differentiating whether respondents live in the “German,” French,” or “Italian” speaking part of Switzerland. Moreover, as migrants are likely to maintain connections with non-resident family members despite often living farther apart (König et al., 2021), they may have developed effective strategies for bridging distances and staying in contact through the use of modern technologies even before the pandemic. Therefore, we also considered cultural differences resulting from migration and included a variable indicating whether respondents were born in Switzerland (“yes” or “no”).

Since the data used in this study were primarily collected during the pandemic, we incorporated various factors that could potentially influence internet behavior in later life. For instance, we examined extensive social distancing situations, indicating whether respondents had never left their home throughout the last 3 months preceding the interview (“yes” or “no”). Additionally, we considered whether respondents or individuals close to them had tested positive for the coronavirus between the first and second COVID-19 surveys which encompassed the period between summer 2020 and 2021. These variables were coded binarily (as “yes” or “no” answers) and captured different situations that could potentially impact internet usage during the pandemic.

In addition to sociodemographic factors, living arrangements, and pandemic-related circumstances, individuals’ attitudes toward and experiences with technology and ICT play a crucial in their current internet usage (König et al., 2018). To assess respondents’ attitudes toward technology, we included four statements: “Technical progress must always go further,” “I could no longer imagine my life without technical devices,” “The increasing digitization has more advantages than disadvantages for society,” and “Robots should be used to care for the elderly.” Response options ranged from “do not agree at all” (1) to “totally agree” (5). In further analyses, these four items were combined using a summative scale score and calculated as the mean of the items ranging from 1 to 5. This scale was labeled as attitudes toward technology (Cronbach’s alpha = 0.67). For models investigating internet intensity, we also included respondents’ internet experience, which was measured in continuous years of past internet usage. Table 1 provides an overview of the descriptive distributions of all independent variables included in our main analysis.

**Table 1:** Sample characteristics.

	Min	Max	Mean/Proportion	SD
Age	50.00	96.00	72.51	8.05
Men			46.97%	
Education				
Low			14.11%	
Medium			65.06%	
High			20.83%	
Employed			18.42%	
Income				
With great/some difficulty			7.63%	
Fairly easily			29.54%	
Easily			62.82%	
Health				
Excellent			8.80%	
Very good			31.70%	
Good			42.57&	
Fair/poor			16.93%	
CASP–12	17.00	48.00	40.39	4.96
Big Five: Openness	1.00	5.00	3.70	0.91
Big Five: Conscientiousness	1.50	5.00	4.27	0.72
Big Five: Extraversion	1.00	5.00	3.51	0.96
Big Five: Agreeableness	1.00	5.00	3.68	0.78
Big Five: Neuroticism	1.00	5.00	2.45	0.94
Living alone			26.56%	
Urban area of residence			22.57%	
Language region				
German			73.78%	
French			23.32%	
Italian			2.90%	
Migrant			13.61%	
Never left home			4.12%	
COVID–19 disease: Respondent			5.02%	
COVID–19 disease: Someone close			43.79%	
Attitudes toward technology	1.00	5.00	3.01	0.82
Internet experience (years)	0.00	30.00	18.65	7.36

Notes: SD stands for standard deviation. Source: SHARE Waves 8 and 9, Swiss subsample, release 8.0.0, unweighted, own calculations.

### 6.4. Analytical strategy

Based on the Swiss drop-off from 2021 and merged with information from the second COVID-19 survey conducted shortly before, our main sample consists of 1,560 respondents. Out of this, we excluded all respondents who are younger than 50 years old (*n* = 6) and those residing in nursing homes (*n* = 10). We also had to exclude 112 interviews that had at least one missing value in one of our dependent variables, including 12participants with seemingly wrong information regarding their average online activity, which exceeds the possible daily limit. The same applied to 227 respondents who had missing information in one of the explanatory variables used. Considering these exclusions, our main sample included 1,205 complete interviews investigating the different patterns of internet usage among respondents in the later stages of their lives living in Switzerland. Given the different dependent variables, our multivariate analyses were based on logistic (ever and recent use), ordinal (internet frequency), and linear (internet intensity) regressions. Before beginning our analysis, we provided a descriptive overview of the trend of recent internet use between 2011 and 2021 based on information on respondents from previous SHARE Waves aged 50 years or older who did not live in nursing homes. Moreover, based on our main sample, we also presented insights regarding different types of internet use and experiences by gender. An overview of our analytical strategy can be found in a simplified representation in the Supplementary Table S1.

## 7. Results

### 7.1. Trends in internet use

**Figure 1:**
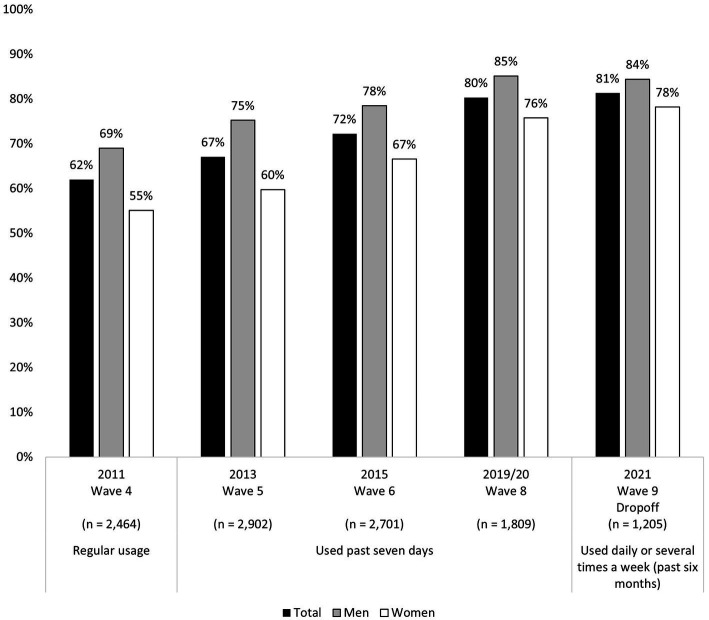
Internet usage in old age across Switzerland over time and by gender. Source: SHARE waves 4, 5, 6, 8, and 9, release 8.0.0, weighted proportions, own calculations.

At a first glance at Figure 1, the level of recent internet use among older adults in Switzerland shows that the majority of residents who are at least 50 years old are online. Simultaneously, our results reveal a continuous and gradual increase in this number over time. While 62% of those surveyed in 2011 were regularly online, 10 years later, this proportion increased to around 81%. Furthermore, we found evidence for both the persistence of gender differences regarding internet use and their decrease over time. In 2021, the gender gap in internet use was around 14%, but by the summer of 2021, it had declined to 6%, indicating that more women have become aware of the advantages the internet has to offer. However, the differences between women and men remain significant over time and in all observed periods (chi-squared test with *p* ≤ 0.010). At the same time, the results indicate that the internet usage among men appears to have plateaued, with around one-sixth not using the internet regularly.

### 7.2. Internet usage, frequency, and intensity: an overview

**Figure 2:**
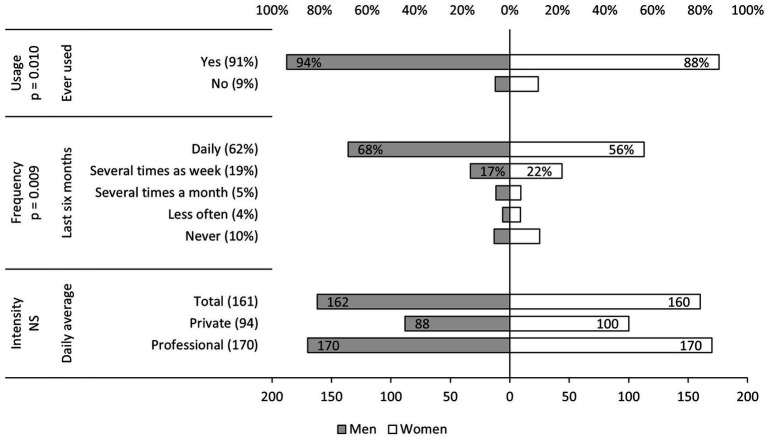
Internet usage, frequency, and intensity in old age across Switzerland during the COVID-19 pandemic (2021) organized by gender. Source: SHARE Wave 9, release 8.0.0, *n* (usage and frequency) = 1,205 (566 men, 639 women), *n* (intensity: total) = 975 (472 men, 503 women), *n* (intensity: private) = 972 (470 men, 502 women), *n* (intensity: professional) = 259 (130 men, 129 women), weighted proportions (usage and frequency) and minutes (intensity), NS stands for not significant, own calculations.

Based on the previous findings, we expanded the analyses of internet use across various domains: Usage, frequency, and intensity (Figure 2). The distributions indicated that approximately one in 10 individuals (9%) had never used the internet in their life. Regarding gender differences, we observed that men have significantly more experience with the internet. While 12% of surveyed women reported never having used the internet in the past, this percentage was only half as large (6%) for men. Among those who were online within the last 6 months, most of them were highly active internet users. Specifically, 62% reported being online every day, and an additional 19% reported being online several times a week. Similarly, gender-specific patterns emerged regarding internet frequency, which showed that men used the internet on a daily basis more than women (68% vs. 56%). In terms of internet intensity, we found that Swiss residents aged 50 years and older spend an average of about two and a half hours (161 min) online per day. Moreover, when differentiating between private and professional internet use, we observed that individuals spend more time online for professional reasons (170 min) than for private purposes (94 min). While general internet use and frequency vary by gender, there are no significant differences between men and women in terms of average use (intensity).

### 7.3. Internet experience and COVID-19

**Figure 3:**
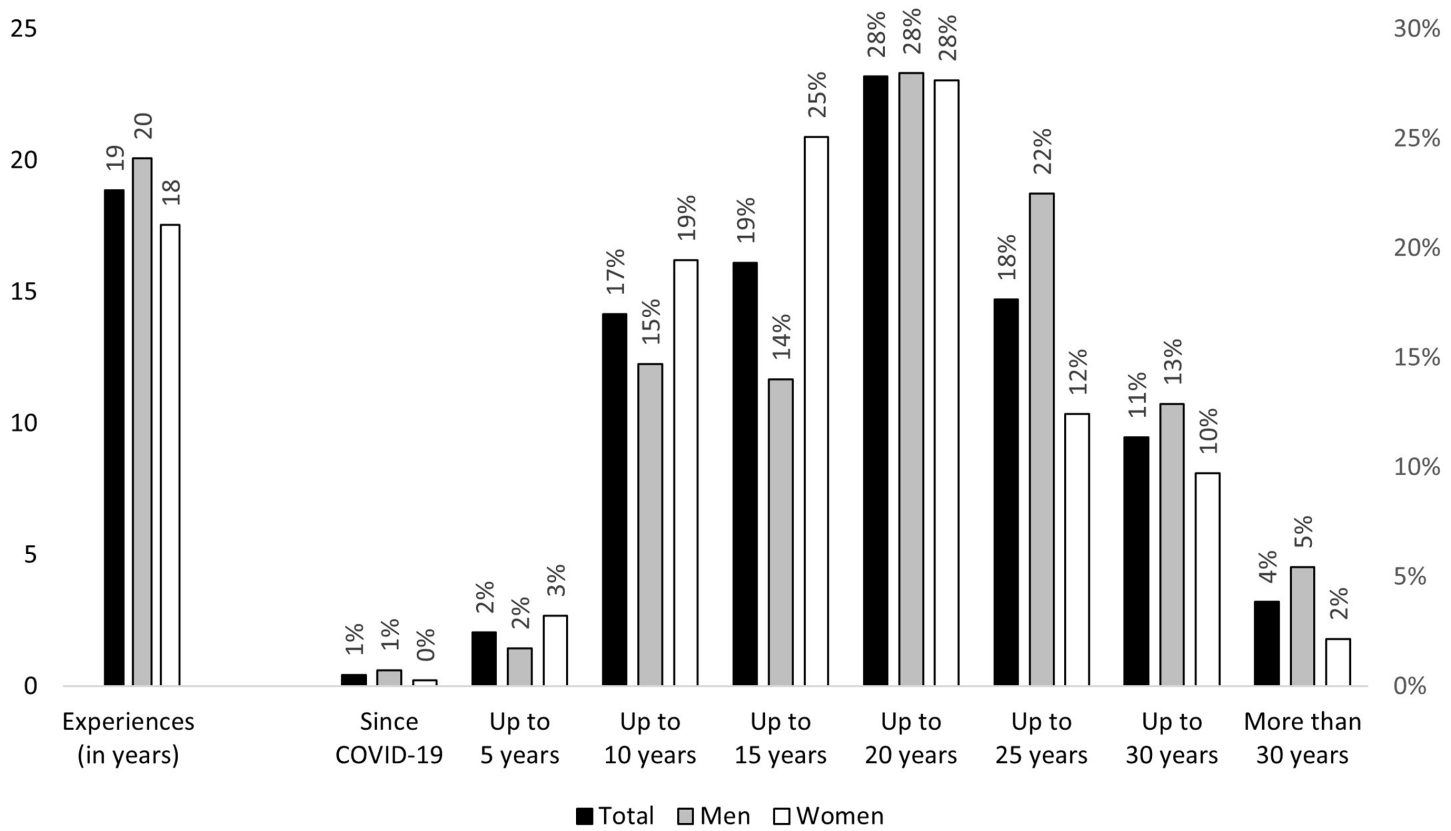
Internet experience in old age across Switzerland by gender. Source: SHARE Wave 9, release 8.0.0, *n* = 980 (503 women, 477 men), weighted, own calculations.

In addition to the previously reported recent internet behavior of Swiss residents in the later stages of their lives, the duration and experience of individuals who used the internet could play a decisive role in their current usage behavior. Based on the question “For how many years have you been using the Internet?,” it can be observed (Figure 3) that older people in Switzerland have been using the internet for an average of around 19 years. In addition to more frequent internet use, men also have on average 2 years more experience using the internet compared to women (20 years vs. 18 years, t test significant with *p* = 0.000). Regarding the influence of the COVID-19 pandemic as a potential push factor for starting to use the internet, the findings in Figure 3 clearly indicate that, at least in Switzerland, this was not the case. Less than 1 % of recent internet users reported starting to use the internet in 2020 or 2021. In fact, over 80% had been using the internet for over a decade. However, Switzerland already demonstrates a high level of internet diffusion among older people (Figure 1), both in comparison to other European countries (König et al., 2018; König and Seifert, 2020) and in general, which reduces the proportion of older individuals who have yet to start using the internet both in regular circumstances and in response to the pandemic.

### 7.4. Determinants for internet use, frequency, and intensity

To analyze and compare the factors influencing different patterns of internet use in later life among Swiss residents, we estimated several models considering individual characteristics, living arrangements, and pandemic-related circumstances. Additionally, attitudes and, if applicable, experiences regarding ICT were included in additional models. Table 2 presents the multivariate results for five different measures of internet use. Logistic regressions were used for never use and recent use, while ordinal regressions were employed to assess internet frequency. Linear regression analyses were applied to the models investigating internet intensity (total and private), where the average daily usage was converted to logarithmic minutes beforehand.

**Table 2:** Patterns of internet usage, frequency, and intensity in Europe.

	Never used		Used in the last 6 months		Frequency		Intensity (total)		Intensity (private)	
	OR	OR	OR	OR	R	R	R	R	R	R
Age	1.14***	1.15***	0.90***	0.90***	−0.06***	−0.06***	−0.01**	−0.01	−0.01	−0.01
Men	0.65	0.65	1.46*	1.46*	0.21**	0.20**	0.07	−0.01	−0.01	−0.07
Education (Ref.: Low)										
Medium	0.35***	0.34***	1.82**	1.81**	0.33**	0.32**	−0.03	−0.01	−0.05	−0.04
High	0.11***	0.11***	4.05***	3.95***	0.80***	0.77***	0.19	0.14	0.17	0.13
Employed	0.38	0.37	1.33	1.36	0.09	0.12	0.60***	0.62***	−0.22**	−0.20*
Income (Ref.: With great/some difficulty)										
Fairly easily	0.94	0.89	0.86	0.93	0.02	0.06	−0.28*	−0.26*	−0.38**	−0.36**
Easily	0.93	0.92	1.10	1.13	0.09	0.09	−0.31*	−0.35**	−0.40**	−0.43***
Health (Ref.: Excellent)										
Very good	1.06	0.96	1.03	1.04	−0.11	−0.09	0.09	0.07	0.06	0.05
Good	2.44	2.09	0.69	0.74	−0.25	−0.22	0.12	0.09	0.11	0.09
Fair/poor	2.17	1.80	0.67	0.72	−0.31	−0.28	0.02	0.02	−0.03	−0.03
CASP–12	0.94*	0.95*	1.05**	1.04*	0.03**	0.02**	0.01	0.01	0.01	−0.01
Big Five: Openness	0.79*	0.79*	1.27**	1.26**	0.13***	0.12**	0.03	0.02	0.01	0.01
Big Five: Conscientiousness	1.16	1.07	0.88	0.92	−0.11	−0.09	−0.09*	−0.09*	−0.13***	−0.12***
Big Five: Extraversion	0.93	0.92	1.03	1.03	0.01	0.01	0.02	0.03	0.03	0.04
Big Five: Agreeableness	0.91	0.92	0.98	0.98	−0.02	−0.01	−0.02	−0.01	−0.04	−0.03
Big Five: Neuroticism	1.07	1.01	0.93	0.97	−0.04	−0.02	−0.02	0.01	−0.01	0.01
Living alone	0.92	0.85	0.96	0.99	−0.02	−0.02	0.18**	0.15**	0.17**	0.14**
Urban area of residence	1.09	1.26	1.11	1.01	0.01	−0.03	0.11	0.09	0.09	0.08
Language region (Ref.: German)										
French	0.81	0.96	1.08	0.93	0.09	−0.01	−0.02	−0.04	−0.01	−0.02
Italian	3.06*	3.20*	0.53	0.53	−0.35	−0.35	0.18	0.24	−0.09	−0.03
Migrant	0.97	1.00	0.89	0.82	0.15	0.09	0.21**	0.17**	0.10	0.07
Never left home	/	/	1.02	0.99	0.12	0.11	0.10	0.06	−0.04	−0.08
COVID–19 disease: Respondent	/	/	0.69	0.74	0.01	0.05	−0.12	−0.13	−0.04	−0.05
COVID–19 disease: Someone close	/	/	1.52**	1.53**	0.19**	0.19**	−0.03	−0.03	−0.04	−0.04
Attitudes toward technology	/	0.44***	/	1.85***	/	0.39***	/	0.13***	/	0.12***
Internet experience (years)	/	/	/	/	/	/	/	0.03***	/	0.02***
*N*	1,205	1,205	1,205	1,205	1,205	1,205	975	975	972	972
*R^2^*	0.28	0.33	0.20	0.23	0.12	0.14	0.18	0.24	0.07	0.14

Notes: Logistic (“never used” and “used last 6 months”), ordinal (“frequency”), and linear (“intensity”; total and private) regressions. Odds ratios (OR) and regression coefficients (R) displayed; robust standard errors; / stands for not applicable; significance levels: *** *p* ≤ 0.001, ** *p* ≤ 0.010, * *p* ≤ 0.050. Source: SHARE Waves 8 and 9, release 8.0.0, unweighted, own calculations.

In general, the results confirmed previous findings that ICT usage, particular internet behavior, is strongly associated with cohort or age. Specifically, older Swiss residents are more likely to have no prior experience with the internet. Furthermore, recent internet use and frequency are negatively correlated with age. However, when considering respondents’ experience with the internet, the average daily intensity of being online does not vary by age. In other words, age does not affect internet intensity if individuals had early experiences with the internet. The often-discussed gender gap in internet behavior (Mariscal et al., 2019) was partially confirmed in our analyses, as men were more likely to have recent and frequent internet use compared to women. However, there were no gender-specific differences in terms of overall and daily usage. While the latter finding aligns with our descriptive results (Figure 2), the higher proportion of non-users among women becomes less significant when accounting for respondents’ education, indicating the importance of higher educational credentials in determining internet usage experiences.

Our findings for education were similar to those for age, as less educated individuals had a higher likelihood of never being online and used the internet less frequently and less often recently. In contrast, employment did not affect internet use and frequency, but it did impact intensity. We found that employed respondents had a higher overall daily internet intensity but were less intense online for private matters. This suggests that many professions in Switzerland require the use of ICT. Like employment, the financial situation only affected average daily usage. Swiss residents whose households were able to make ends meet (fairly) easily used the internet less intensively in their daily lives compared to those reporting some or great financial restrictions.

Considering their various life circumstances, subjective health did not have a direct impact on internet behavior in later life. In contrast, respondents with a higher quality of life, as measured by CASP-12, were not only more likely to have ever used the internet, but they also used it more often and more frequently recently. However, they exhibited lower intensity in their online activities. Regarding the influence of personality on internet use, the findings indicated that individuals with a high level of openness were more likely to be open to technology such as the internet and had more often and frequent experience with it. On the other hand, only conscientious individuals with a high degree of self-control, accuracy, and determination used the internet with significantly less intensity in their everyday lives.

We also observed that living arrangements and structural cultural patterns partly influenced individuals’ internet behaviors. Respondents who lived alone and thus had fewer social interactions in their home environment spent more time online on average. The urbanization of their living area did not have any impact on the examined measures of internet use, indicating that general internet access did not vary between (big) cities and rural areas in Switzerland. Regarding regional differences within Switzerland, we found that respondents aged 50 years and older living in Ticino were more likely to have no experience with the internet at all. For those with internet experience, there were no differences among the three language regions in terms of recent use, frequency, and intensity. However, it is important to note that while the sample size of Italian speaking respondents roughly corresponds to their percentage of the total population (Table 1), the relatively small sample size may lead to potential under-or overestimations of the observed effects. Regarding differences caused by migration, the results highlight that foreign-born residents of Switzerland spend more time using the internet than the native population in the same age group. However, this effect seems to be true for total internet use but not private intensity. This suggests that non-natives are more likely to be employed in jobs involving internet use.

Furthermore, the inclusion of pandemic-related events at the individual level indirectly affected internet use in 2021. Being isolated at home or personally infected by COVID-19 did not appear to have any effect on internet use. However, if someone close to them was infected, it became important. In such cases, respondents were more likely to be online more often and frequently (though not intensely). This could be attributed to their increased need for obtaining pandemic-related information and news as well as staying in contact with family, relatives, and friends through online means.

Finally, when examining attitudes toward technology and internet experience, specific influences on current internet use were observed. In general, higher levels of curiosity and interest in technology were associated with a greater likelihood of having used the internet in the past and currently. Similarly, respondents with a stronger affinity for technology were more frequent and engaged in more intensive online activities. Furthermore, a greater affinity for technology could also be inferred from earlier internet usage, which significantly impacted internet intensity. The longer respondents had been using the internet, the more time they spent online every day. Moreover, including both ICT-related items at the individual level yielded the best model fit for each of the dependent variables used. This emphasized that the use of the internet in the later stages of life strongly depends on one’s own interest in modern technology and one’s personal experiences with it. In other words, technological curiosity accelerates early adoption of the internet and, consequently, its continued use in later life.

## 8. Discussion

This study explored the digitalization processes shaping peoples’ everyday life in the 21st century and their impact on individuals’ development later in life. It highlights the significance of the person-environment fit in successful aging and emphasizes the role of technology in this context. However, older adults often lack access to and proficiency in using new technologies, which can lead to their exclusion from the digitally dominated society (Hunsaker and Hargittai, 2018). The digital divide, particularly regarding internet use, is still present among older adults based on age and cohort groups. Besides basic access, the frequency and intensity of internet use among older adults are important factors to consider. The COVID-19 pandemic has further highlighted the digital divide, with internet use playing a crucial role in enabling older individuals to communicate and access various services (Seifert et al., 2021). However, those who do not use the internet or have limited internet skills may experience social isolation (Robinson et al., 2020). This article presents three research questions to address these issues: the change in the proportion of older internet users over time, the frequency and intensity of internet use during the pandemic, and the predictors of internet use at different levels.

The findings revealed that the majority of Swiss residents aged 50 and above are online, with a continuous increase over time. Gender differences in internet use persist but have diminished over the years, with an increasing number of women becoming aware of the advantages of the internet, as observed in other recent European studies (Bünning et al., 2023). However, differences between men and women remain significant. The study also explored internet usage, frequency, and intensity. Approximately 9% of individuals have never used the internet, with men having more experience than women. Among recent internet users, a majority are highly active, with 62% being online every day. Swiss residents aged 50 and older spend an average of about two and a half hours online per day, with more time spent on professional use than private use. The duration and experience of internet use play a role in current usage behavior, with older people in Switzerland having an average of around 19 years of internet experience. The COVID-19 pandemic did not significantly influence internet adoption among older adults in Switzerland. Current findings from other studies also suggest that Internet use among older individuals has not significantly increased during the pandemic, in contrast to the expected substantial rise over a 1–2-year period (Gallistl et al., 2021). Factors such as age, education, employment, living arrangements, and attitudes toward technology are associated with different aspects of internet use.

In terms of determinants of internet use, higher age is associated with no prior internet experience, lower recent use, and less frequency. These results are in line with previous findings (Olson et al., 2011). However, age does not affect internet intensity if individuals had early experiences with the internet. Men are more likely to have more recent and frequent internet use compared to women, but there are no gender differences in overall and daily usage when accounting for education. A lower education level is associated with a higher likelihood of never being online and less frequent and recent use. Employment is related to higher overall daily internet intensity but less intensity for private matters, suggesting that certain professions in Switzerland require ICT use. Subjective health does not directly impact internet behavior, but a higher quality of life is associated with more frequent and recent internet use. Personality traits, such as openness and conscientiousness, also play a role in influencing internet usage. This aligns with previous research among younger adults, which demonstrated that conscientiousness predicts overall Internet use (Mark and Ganzach, 2014) However, research that also includes older adults (born before 1964) has reported that conscientiousness was a significant predictor of internet usage only for the younger individuals (born after 1965), not for the older adults (Roos and Kazemi, 2021). Living arrangements, urbanization, and regional differences have partial effects on internet behavior. Foreign-born residents spend more time online than the native population, especially for total internet use. Pandemic-related events indirectly affect internet use, with individuals being more online when a close person is infected. Attitudes toward technology and internet experience play a significant role in current internet use, with curiosity, interest, and affinity for technology influencing internet adoption and intensity. Overall, the study highlighted the multifaceted factors that shape internet use among older adults in Switzerland.

Given that this study specifically concentrated on Switzerland, the generalizability of our findings to contexts outside of Switzerland may be limited. Nevertheless, the data from Switzerland can serve as a valuable case study. Although the SHARE data allows us to examine internet usage among older adults in various European countries, there are some important variables that were not included in the survey. These variables include technological biographies, technology acceptance, technology use within households, and the reasons for non-use. Future studies using representative data should aim to investigate the factors influencing changes in internet usage status more comprehensively. This can be achieved using longitudinal studies which provide a deeper understanding of how internet use evolves over time. Furthermore, international data is necessary to gain insights into the digital divide, including whether the divide exists among different age cohorts and/or countries as well as how it develops over time.

However, based on the findings, social policy recommendations to promote internet use among older adults in Switzerland could include targeted initiatives to reduce the gender gap and increase digital literacy, particularly among women (Bünning et al., 2023). Additionally, providing accessible digital education programs for older adults with lower levels of education and addressing the specific needs and preferences of older adults in designing online services and platforms can contribute to enhancing their internet adoption and engagement. An example would be providing specific training with individualized support for older adults. Other studies have demonstrated the moderating effect of technology training and support on the relationship between technology exploration and perceived learning difficulties (Tsai et al., 2019).

## 9. Conclusion

This study examined the digital divide among older adults in Switzerland during a worldwide pandemic. It focused on the proportion of users, frequency, and intensity of use during the COVID-19 pandemic. The findings indicated a growing proportion of internet users over time, although around 9% of individuals involved had never used the internet, while recent users exhibit high activity levels, spending an average of approximately two and a half hours online daily. The study identified age, education, employment, living arrangements, and attitudes toward technology as influential factors determining internet use in 2021. Nevertheless, the COVID-19 pandemic did not have a significant impact on internet adoption among older adults, showing that there was not a big “digital push” among this segment of the population. These findings shed light on the complex dynamics that shape internet use among older adults and underscore the need to promote intervention to aid in the digital inclusion of this segment of the population.

## Data availability statement

Publicly available datasets were analyzed in this study. This data can be found at: the datasets analyzed for this study can be found in the SHARE Research Data Center (https://share-eric.eu/data/).

## Author contributions

RK: Writing – original draft, Writing – review & editing. AS: Writing – original draft, Writing – review & editing.
