# Supplementary material for: Internet usage, frequency and intensity in old age during the COVID-19 pandemic—a case study for Switzerland
Source: Front Sociol. 2023 Oct 26;8:1268613. doi: 10.3389/fsoc.2023.1268613 (PMC10639129; doi:10.3389/fsoc.2023.1268613)
Supplement: Supplementary file 1 [file Table_1.DOCX]

Supplementary Material

**Table S1.** Analytical strategy

|  | Additional data | | | | | | | Main data | |
| --- | --- | --- | --- | --- | --- | --- | --- | --- | --- |
|  | Wave 4 | | Wave 5 | | | Wave 6 | Wave 8 | Dropoff questionnaire | |
|  | 2011 | | 2013 | | | 2015 | 2019/20 | 2021 | |
|  |  | |  | | |  |  | Version A | Version B |
|  |  | |  | | |  |  |  | |
| **Measurements:** |  | |  | | |  |  |  | |
|  |  | |  | | |  |  |  | |
| 1. Ever used |  | |  | | |  |  | **✓** | |
| 1. Recent use | **✓** | | **✓** | | | **✓** | **✓** | **✓** | |
| 1. Frequency |  | |  | | |  |  | **✓** | |
| 1. Intensity |  | |  | | |  |  |  |  |
| Total |  | |  | | |  |  | **✓** |  |
| Private |  | |  | | |  |  | **✓** |  |
|  |  |  | |  |  | | |  |  |
| **Analyses:** |  |  | |  |  | | |  |  |
|  |  | | | | | | | | |
|  | Variations in recent internet use over time (descriptive) | | | | | | | | |
|  |  |  | |  |  | | |  |  |
|  |  |  | |  |  | | | Variations in internet use by groups (descriptive) | |
|  |  |  | |  |  | | |  |  |
|  |  |  | |  |  | | | Patterns for measurements 1, 2, and 3 (multivariate) | |
|  |  |  | |  |  | | |  |  |
|  |  |  | |  |  | | | Patterns for measurement 4 (multivariate) |  |
|  |  |  | |  |  | | |  |  |

Notes: Own representation.
